# Pessary with perineal suture for treatment of pelvic organ prolapse: description and benefit of the technique

**DOI:** 10.1007/s00404-022-06739-2

**Published:** 2022-10-15

**Authors:** Marta Pérez-Febles, Sonia De-Miguel-Manso, Elena García-García, María López-País, María Cuaresma-González, Marta Ibañez-Nieto

**Affiliations:** 1 Servicio de Obstetricia Y Ginecología, Hospital Clínico Universitario de Valladolid, Gerencia Regional de Salud de Castilla Y León (SACYL), Avenida Ramón Y Cajal 3, 47005 Valladolid, Spain; 2 Departamento de Pediatría E Inmunología, Facultad de Medicina, Obstetricia Y Ginecología, Nutrición yBromatologíaUniversidad de Valladolid, Psiquiatría e Historia de la Ciencia, Valladolid, Spain

**Keywords:** POP, Pessary, Perineal suture

## Abstract

**Introduction and hypothesis:**

Vaginal pessaries are used as a conservative treatment for POP in women who do not want or are not candidates for surgery, or as a preliminary step to surgery. Our goals are: evaluate the evolution of patients with advanced POP and repeated expulsion of the pessary, who underwent perineal suture to try to maintain the device. Describe the epidemiological characteristics of patients treated with pessaries in our environment, with or without perineal closure.

**Methods:**

Observational, descriptive and prospective study (October 2016-March 2021) that includes 352 women with advanced-stage POP treated with a pessary, of which 55, after repeated expulsion of the pessary, were treated with a pessary and perineal suture.

**Results:**

After pessary insertion associated with perineal closure, 26 patients (47.2%) expelled the pessary and underwent surgery, and 29 (52.8%) kept the device, avoiding surgery. Regarding the women who required perineal suture: The mean age was higher than in the group of patients who did not need this intervention (75.3 vs. 68.3 years), 94.5% had POP ≥ grade III and 100% had a perineal width > 2.5 cm.

**Conclusions:**

Treatment with pessary and perineal closure avoids surgery in women with advanced age and repeated expulsion. Although age should not be an independent factor that limits surgical treatment or the type of intervention, it would be useful to have scales to quantify the frailty of patients, being able to standardize perineal closure in elderly and/or frail women, and in those who do not want or have contraindications for surgery.

## What does this study add to the clinical work

**Table:** 

Perineal suture, as a treatment for pelvic organ prolapse, can be of great benefit to very elderly patients or frail patients, since it avoids surgery and improves their quality of life.	

## Introduction

Pelvic organ prolapse (POP) is the descent of different pelvic organs from their normal anatomical position, secondary to failure of the support structures. The most specific and most correlated symptom is the sensation of a lump in the genitals. It is a common problem in women, approximately 40% of them will experience prolapse throughout their lives, and the proportion is expected to increase due to the aging of the population [1].

Vaginal pessaries have been offered as a conservative treatment of POP, mainly to women who will look for a new pregnancy (as a temporary measure), and for those who are not good candidates for surgery or do not wish to undergo POP surgery. It is estimated that almost two-thirds of women with symptomatic POP choose the pessary as initial treatment [2].

There is a relief of prolapse, urinary and intestinal symptoms, with the pessary use, resulting in improved quality of life. Complications are usually mild [3]: increased vaginal discharge (the more common), or vaginal erosion [4] which can be successfully treated with targeted therapy, such as vaginal estrogen supplementation.

Women with a history of hysterectomy [5] or pelvic reconstructive surgery, with posterior vaginal wall prolapse, or under 65 years [6], are more likely to be intolerant to the pessary after initial insertion [4], either because of their expulsion or because of pain. However, we have not found bibliographical references regarding the degree of prolapse or the width of the perineal opening as possible risk factors for expulsion of the pessary.

In clinical practice, our subjective impression is that there is a greater risk of expulsion of the pessary when the POP is very advanced and/or the width of the perineum is greater than 2.5 cm.

## Objectives

The objective of this work was to evaluate the evolution of patients with advanced POP who underwent perineal suture to avoid expulsion of the pessary, and in what way this intervention was effective.

As secondary objective, we proposed to describe the epidemiological characteristics of the patients: on the one hand, those patients who required perineal closure, and on the other hand those who did not. The first group, in turn, was divided into two: the group of patients in whom the intervention was successful and the one in which it failed.

## Materials and methods

This is an observational, descriptive and prospective study of women with advanced pelvic prolapse and persistent expulsion of the pessary, who were offered a perineum suture to maintain the pessary, either as a primary treatment or as a step prior to surgery, achieving better tissue conditions after temporary correction of the prolapse with the pessary.

Depending on the subsequent evolution, we differentiated two groups:

- Group 1: patients who, despite the intervention, expelled the pessary and underwent surgery.
- Group 2: patients in whom the intervention was successful and decided to continue with the device or request surgery.

The study has been carried out in the Pelvic Floor Gynecological Unit of the University Clinical Hospital of Valladolid during the period of time between October 2016 and March 2021 (54 months). Patients are referred to this unit from the gynecological consultations of the hospital's health area for various reasons, mainly due to pelvic organ prolapse and urinary incontinence, but also due to chronic pelvic pain, anal incontinence and obstetric anal sphincter tears.

After physical examination, prolapse staging was performed using Baden–Walker classification, in grades I, II, III and IV [7].

During that period, only silicone regular or thick ring pessaries were used and the perineum sutures were performed in the same medical consultation. The patient was informed of this option, to achieve a temporary closure of the perineum and thus try to keep the pessary inside the vagina and verbal consent was obtained.

Regarding the approval by the Ethical/Institutional Review Board: this study did not require approval. Verbal consent was obtained from the patient in consultation after detailed information.

**Fig. 1:**
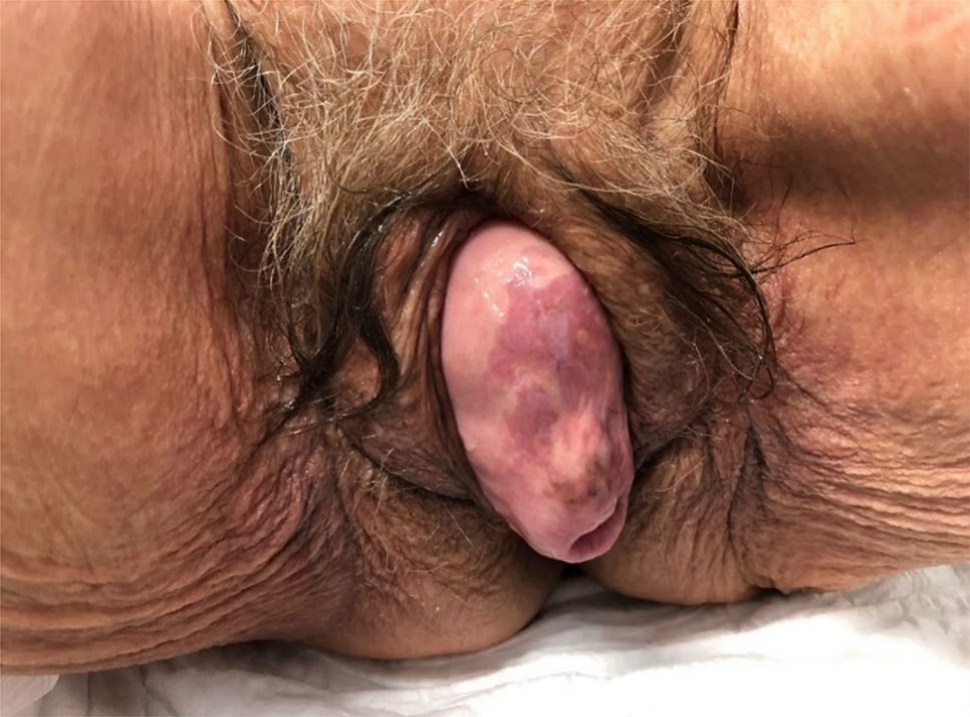
Grade IV anterior and middle compartment prolapse with superficial ulceration of areas of the vaginal mucosa of patient 1

**Fig. 2:**
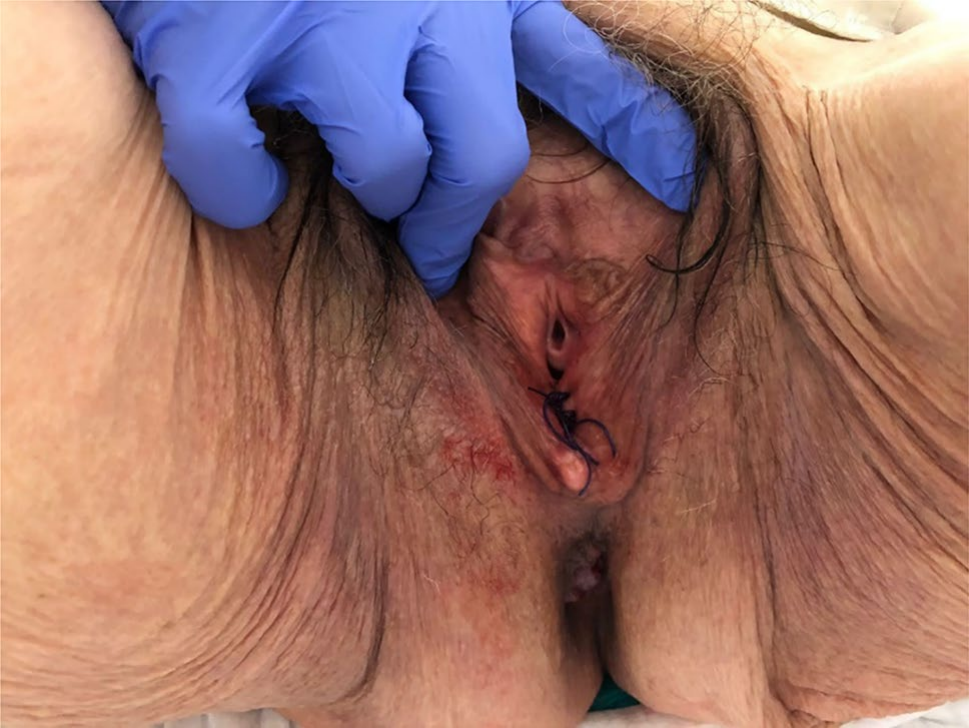
Result after insertion of pessary with 3 absorbable sutures in the perineum of patient 1

**Fig. 3:**
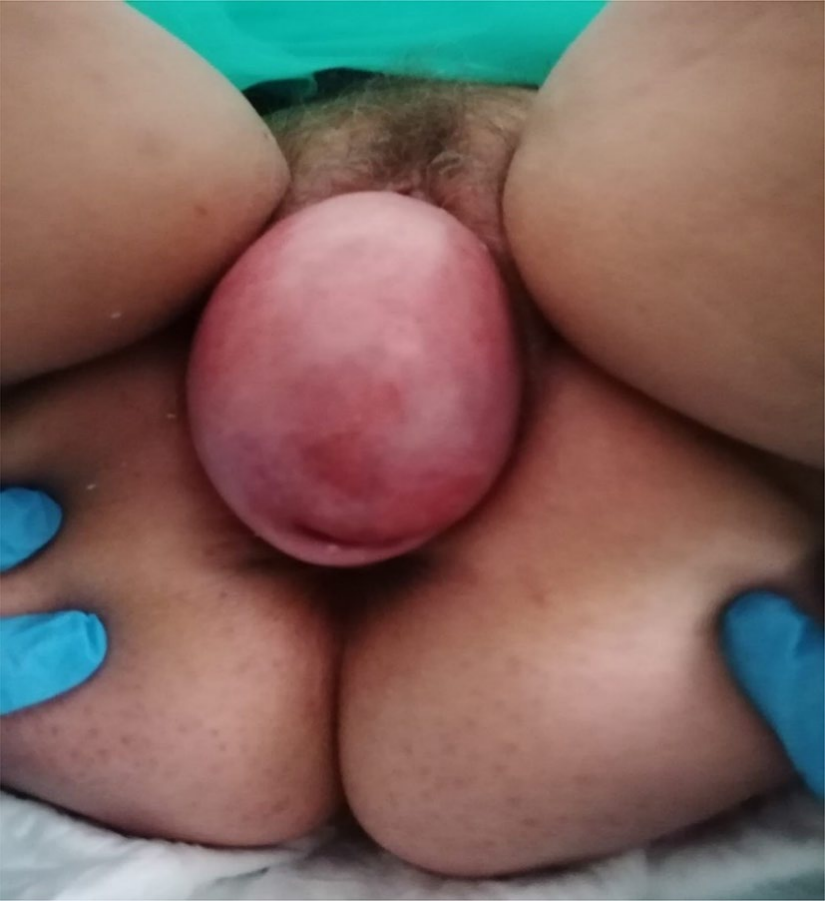
Total eversion of the vagina with superficial ulceration of the anterior lip of the cervix of patient 2

**Fig. 4:**
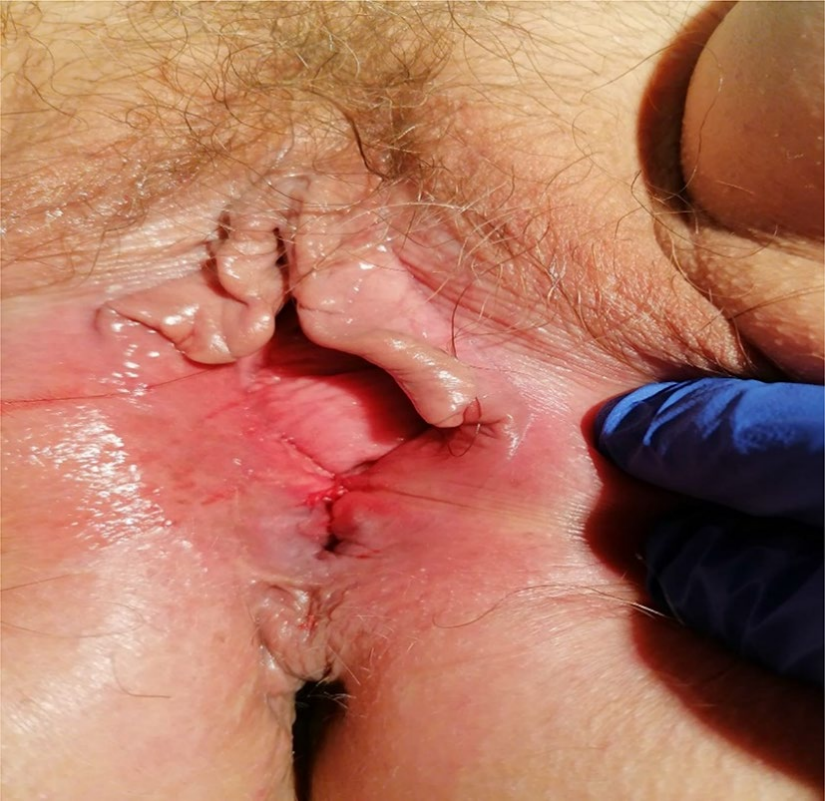
Result after insertion of pessary with placement of sutures in the perineum of patient 2

After ruling out allergies to local anesthetics and anticoagulant treatments, the perineum was sutured under asepsis with aqueous chlorhexidine and local anesthetic infiltration, with 3 independent sutures of resorbable USP braided multifilar polyglycolic acid 1 Safyl® Violet (B. Braun Melsungen AG Germany). This suture maintains tension and therefore closure of the introitus for 18 days in 50% of cases, with complete absorption at 60 days. Figures 1,2,3,4 show 2 examples of advanced POP and excessive perineal opening, before and after pessary insertion with perineal closure with sutures.

Description of the technique: first, the insertion of the pessary was performed, infiltration of local anesthetic. Subsequently, two or three loose stitches were given, depending on the patient’s perineal opening (> 2.5 cm), reinforcing the perineal body, achieving reduction of the introitus and increased tension of the vaginal canal, without performing colpotomy. It is not necessary to perform the perineum stitches with each change of the pessary.

Subsequent recommendations: usual analgesia for 24 or 48 h if required.

The first follow-up visit is a month, where it is assessed whether the adjustment has been carried out successfully, if so, a weekly chlorherxidine suppository is recommended.

Most of these patients are older, do not want to remove, wash and place the pessary by themselves, and the pessary is changed in a period of 4 to 6 months in gynaecology clinics.

## Results

In this time interval, 838 patients were referred to the Pelvic Floor Unit, 466 were referred for POP, 297 received first-line treatment with pessary (63.7%), and 55 women presented advanced POP with repeated expulsion there of (6 0.6%), so they were offered perineum suture after pessary insertion. 53 (17.8%) patients with pessary insertion without perineum sutures finally underwent POP surgery (Table 1).

**Table 1:** Patients referred to the Pelvic Floor clinic for POP and treated primarily with a pessary, with and without perineal suture

Patients referred to pelvic floor consultation for POP (*N*)	466
Patients with pessary without perineum sutures *N* (%) Surgery for pessary treatment failure	297 (63,7) 53 (17,8)
Patients with pessary with perineum sutures *N* (%) Group 1: expulsion and surgery Group 2: pessary maintenance	55 (6,6) 26 (47,2) 29 (52,8)

In patients with perineum sutures, 94.5% presented POP ≥ grade III and 100% had perineum width greater than 2.5 cm.

The types of POP were: 38.2% anterior and middle compartment, 25.4% anterior compartment, 21.8% middle (uterine) compartment, 12.7% vaginal vault prolapse, and 1.8% posterior compartment prolapse.

The most common symptoms associated with POP in these women with an advanced-stage and repeated expulsion of the pessary were urinary: 14.5% of the patients reported urgency, 12.7% urgency urinary incontinence (UUI), 10.9% % stress urinary incontinence (SUI), and only 1.8% had voiding dysfunction.

After inserting the pessary associated with the perineum sutures (55), 26 patients (47.2%) expelled the pessary and had to undergo surgery, while 29 (52.8%) patients successfully maintained the pessary during the study period (Table 1).

If we add all the patients who, after pessary insertion, received surgery for its complications, with or without perineal sutures, the figure rises to 79 (22.4%).

When studying the epidemiological characteristics of the sample, we observed that the mean age of the patients with pessaries without sutures on the perineum was 68.6 years, compared to 75.3 years of patients with sutures on the perineum. In group 1 of the patients with perineum sutures, the mean age was 75.9 and in group 2, 74 years (Table 2).

**Table 2:** Description of the mean age and BMI of the patients treated with a pessary with and without perineal suture

	Average age	IMC
Pessary without perineum sutures	68.6 years	26,2
Pessary with perineum sutures Group 1 Group 2	75.3 years 75.9 years 74 years	25,5 26,6 24,5

The mean body mass index (BMI) in patients with a pessary and without perineum sutures was 26.2, while in those who received perineal suture, it was 25.5, being 26.6 in group 1 (grade I overweight) and 24.5 in group 2 (normal weight) (Table 2).

If we assess the associated comorbidities in the group of patients with perineum sutures, we find arterial hypertension in 69.1% of them (76.9% in group 1 and 62% in group 2), cardiological pathology in 29, 1%, dyslipidemia in 27.2%, type 2 diabetes mellitus in 23.6%, thyroid disease in 10.9%, oncological disease in 7.3%, respiratory disease (asthma) in 5.4%, cognitive impairment in 3.6%, and vascular pathology in 1.8%.

3.6% of the patients in the group with perineum sutures died during this time interval.

The average time elapsed between pessary insertion with the perineum sutures and surgical intervention due to its failure was 7 months, with a minimum of 1 month and a maximum of 36 months, in a single case.

**Fig. 5:**
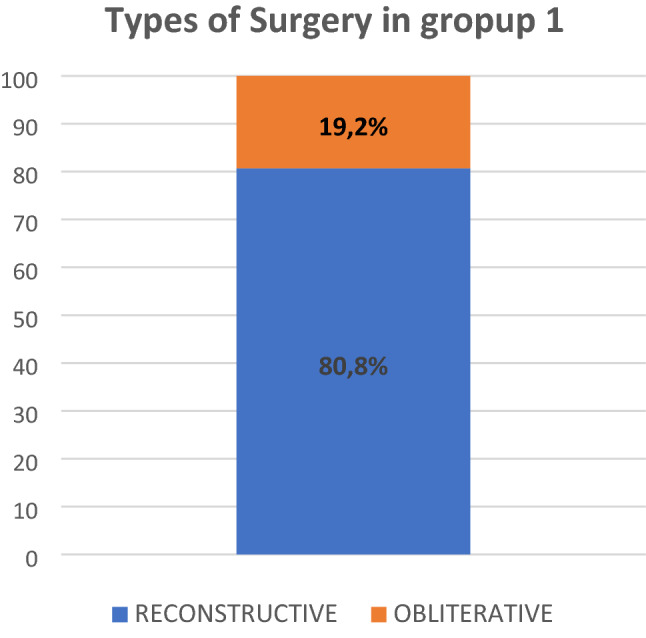
Distribution of the type of surgery (reconstructive versus obliterative) in group 1 patients, who expelled the pessary despite the perineal stitches

**Fig. 6:**
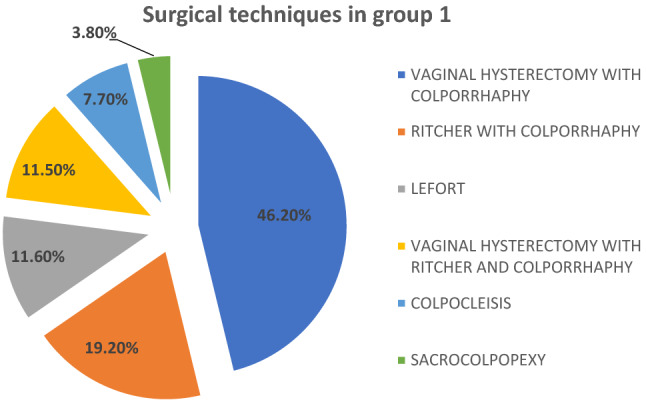
Surgical techniques performed in group 1 patients

The types of surgical intervention to which the patients in group 1 underwent (Figs. 5 and 6) were reconstructive techniques in 80.8% and obliterative techniques in 19.2%. Within the restorative surgeries, the most frequent were: vaginal hysterectomy (VH) with anterior and/or posterior colporrhaphy (46.2%), followed by Richter operation with anterior and/or posterior colporrhaphy (19.2%). Sacrocolpopexy was indicated in 3.8% of these patients.

## Discussion

Treatment of POP with a pessary is common, in up to two-thirds of patients with symptomatic POP [2], since it improves quality of life, and its complications, if any, are mild. This has been shown even in advanced POP, reaching high success rates [3, 14].

In our sample, 63.7% of the patients referred for POP received the pessary as first-line therapy. If we add to these patients who received the perineum suture, the percentage increases to 75. 5%. This represents a higher percentage than that described in the literature, since we insist on the non-invasive treatment of women with advanced POP in whom the pessary has failed to avoid surgery (they are usually elderly patients with multiple comorbidities), or schedule a surgical treatment with the vaginal tissues in better conditions.

Despite primary treatment with a pessary (with or without suture), 22.4% of these women required surgery due to its failure (expulsion, complications). This figure would have risen to 30.7% if the perineal suture had not been applied to the 55 patients referred for repeated expulsion of the pessary, since 52.5% of them kept the pessary thanks to this intervention, thus avoiding surgery.

A 2-year follow-up study reported a 31% surgery rate after successful pessary placement in 444 women with POP [15] and a retrospective review of 794 patients reported a 33% rate [4], higher than ours, probably because we offer the perineal suture to patients with repeated expulsion of the pessary and advanced POP.

The mean age of the group of patients with pessary without perineal closure was 68.6 years, lower than that of the group of patients who underwent perineal suture after pessary insertion, which was 75.3 years. This may be due to the fact that older women may present more advanced POP (greater laxity of the tissues), more frequent comorbidities and a longer evolution time, and consequently, a repeated expulsion of the pessary. This is the reason that pushes us to insist on the pessary as a treatment for POP, to try to avoid surgery in this type of women.

Several authors have compared pessary therapy with surgical treatment, in terms of results and complications. A study with a 1-year follow-up did not present significant differences in the improvement of vaginal, intestinal, and urinary symptoms and quality of life [2]. In relation to adverse effects, with pessary, they were found in 31.6%, all of them Grade I according to the Clavien and Dido classification, whereas after surgery the rate of complications was 39%, being Grade I 14.3%, Grade II 10.4%, and Grade III 14.3% [3].

After analyzing by age group, the serious postoperative complications in 27,403 patients (control group of 45–64 years versus the groups of 65–79 years and ≥ 80 years), it was concluded that serious complications after POP surgery they increase in the group of ≥ 80 years, regardless of frailty and other medical-surgical risk factors [8].

Surgery was required in 47.2% of the patients in the perineum suture group, and the most frequent surgical technique was reconstructive, possibly due to the severity of the prolapse and the frequent total eversion of the vagina in these patients.

The comparison of the results after reconstructive surgery of 217 patients, divided into two cohorts according to age (≥ 75 years and < 75 years), with a mean follow-up of 33–36 months, indicates that patients 75 years or older, adequately prepared, experience the same anatomical, quality of life, morbidity, and mortality outcomes as those under 75 years [17].

Regarding the type of surgery, reviewing a database of the American College of Surgeons, perioperative morbidity and mortality have been compared in patients > 75 years who underwent surgical treatment of POP with colpocleisis, vaginal repair or sacrocolpopexy. The results report that colpocleisis entails a shorter surgical time and a shorter hospital stay, without observing significant differences regarding complications between the three techniques. Patients undergoing colpocleisis are older and have more comorbidities [11].

Another study that analyzed patients who underwent surgery for advanced POP, according to age, BMI, and POP stage, found a significantly lower deterioration in quality of life after obliterative versus reconstructive surgery [16].

After the analysis of a retrospective cohort of 12,731 patients undergoing POP surgery, of which 5.3% underwent colpocleisis, it is described how for POP surgery, age is strongly associated with the type of procedure, and fragility with the postoperative complications [12].

A systematic review reports how preoperative frailty is significantly associated with adverse perioperative outcomes in benign gynecological surgery [9].

The problem is that we would need quick and effective validated tools to analyze the frailty of patients with POP and consequently decide on their approach. A prospective comparison has been made to assess the efficacy of an image-based clinical frailty scale (assessed by the patient and the surgeon) against the Fried frailty index (reference in older patients with pelvic floor pathology), concluding that this scale visual is an excellent predictor of frailty compared to the Fried index [10].

In our sample, we did not carry out an objective assessment of the frailty of the patients, but we subjectively observed how patients with advanced POP and repeated expulsion of the pessary seemed more vulnerable, for different reasons in addition to older age, offering in these cases, as a last resort, pessary insertion with perineal closure in consultation.

We should incorporate frailty objective assessment into our decision-making to improve expectations and outcomes among older women considering POP surgery [12, 14].

We consider that age as an independent factor should not limit surgical treatment or the type of intervention (reconstructive versus obliterative). In our sample, we have tried to reduce the number of surgeries using a pessary with perineum closure. When this fails, the most convenient surgical technique is decided individually, considering complete perineal reconstruction with local anesthesia in younger women who want symptomatic treatment, while we would schedule cleisis for older women.

52.8% of the patients with a pessary and perineum suture maintained the same during the study period, presenting a lower age (74 years vs. 75.9 years) and BMI (24.5 vs. 26.6) than patients who expelled the device. A lower BMI has been described as a factor favoring the continued use of the pessary (OR 0.76 [95% CI 0.62–0.93]) [13].

An interesting aspect would be to evaluate why all the patients subjected to perineal closure, after repeated expulsion of the pessary, had a perineal opening greater than 2.5 cm. Two possibilities would be arisen: the first that it was a risk factor for advanced POP and the second that this perineal width was caused by the magnitude of the POP.

In a prospective observational study of 255 pessary-treated women, avulsion of the levator ani muscle, diagnosed by 3D/4D transperineal ultrasound, was associated with a threefold increased risk of pessary expulsion at 1 year [18].

In our sample, examination of the levator ani muscle (clinical or ultrasound) has not been performed routinely, so we have no data to relate it to the repeated expulsion of the pessary.

## Conclusions

- In our setting, treatment with pessary and perineal closure in consultation has avoided surgery for POP in women with advanced age and repeated expulsion.
- Neither the indication nor the type of surgical treatment for POP should be conditioned by age, preparing patients in the most appropriate way.
- It would be very positive to have validated and easy-to-use scales in consultation to quantify the frailty of patients, since it seems to be a risk factor for surgical complications.
- The perineal suture could be standardized to maintain the pessary in very old (> 80 years) and/or frail women, and in those who do not want surgery or have contraindications for it.

## Abbreviations

POP: Pelvic organ prolapse
BMI: Body mass index
